# Sulfate Deficiency-Responsive MicroRNAs in Tomato Uncover an Expanded and Functionally Integrated Regulatory Network

**DOI:** 10.3390/ijms26178392

**Published:** 2025-08-29

**Authors:** Diego Landaeta-Sepúlveda, Nathan R. Johnson, Jonathan Morales-Espinoza, Mariola Tobar, Evelyn Sánchez, José D. Fernández, Consuelo Olivares-Yáñez, Joaquín Medina, Javier Canales, Elena A. Vidal

**Affiliations:** 1 Centro de Genómica y Bioinformática, Universidad Mayor, Santiago 8580745, Chile; diego.landaeta@mayor.cl (D.L.-S.); nathan.johnson@umayor.cl (N.R.J.); jon.moralese@gmail.com (J.M.-E.); mptobar18@gmail.com (M.T.); evelyn.sanchez@mayor.cl (E.S.); jose.fernandezpe@mayor.cl (J.D.F.); consuelo.olivares@umayor.cl (C.O.-Y.); 2 Agencia Nacional de Investigación y Desarrollo-Millennium Institute for Integrative Biology (iBio), Santiago 8331150, Chile; javier.canales@uach.cl; 3 Agencia Nacional de Investigación y Desarrollo-Millennium Nucleus in Data Science for Plant Resilience (Phytolearning), Santiago 8370186, Chile; 4 Escuela de Biotecnología, Facultad de Ciencias, Ingeniería y Tecnología, Universidad Mayor, Santiago 8580745, Chile; 5 Centro de Biotecnología y Genómica de Plantas, Instituto Nacional de Investigación y Tecnología Agraria y Alimentaria, Consejo Superior de Investigaciones Científicas, Universidad Politécnica de Madrid, 28223 Madrid, Spain; medina.joaquin@inia.csic.es; 6 Instituto de Bioquímica y Microbiología, Facultad de Ciencias, Universidad Austral de Chile, Valdivia 5110566, Chile

**Keywords:** microRNAs, sulfur, sulfate deficiency, tomato

## Abstract

Sulfate availability critically influences plant growth, yet the role of small RNAs, particularly microRNAs (miRNAs), in regulating responses to sulfate deficiency remains poorly understood. Here, we conducted a temporal analysis of sulfate deficiency-responsive miRNAs in the roots and leaves of *Solanum lycopersicum* (tomato), using an updated miRNA annotation in the SL4.0 genome. We found 40 differentially expressed miRNAs, including 2 novel, tomato-specific miRNAs. Tomato miRNAs showed an important time- and organ-specific regulation, similar to the described response of the mRNA transcriptome. Integration with transcriptomic data and Degradome-seq analysis highlighted both canonical and non-canonical targets for sulfate-responsive miRNAs. miR395, the most extensively studied miRNA, was found to control not only its conserved targets involved in sulfate transport and assimilation, but also genes involved in redox homeostasis, photosynthesis and chloride transport. Notably, most targets were repressed in leaves, suggesting miRNA-mediated downregulation of energy-intensive processes, while root targets were predominantly upregulated, including genes related to protein remodeling and antioxidant defense. Comparative analysis with *Arabidopsis thaliana* revealed a broader functional repertoire in tomato, suggesting species-specific adaptations to sulfate deficiency. Overall, our results underscore the critical role of miRNAs in fine-tuning organ-specific metabolic reprogramming during nutrient stress, expanding the current understanding of the regulatory landscape underlying sulfate deficiency in plants.

## 1. Introduction

Sulfur (S) is an essential nutrient for all living organisms, serving as a key constituent of numerous biomolecules including the amino acids cysteine and methionine, glutathione (GSH), vitamins, cofactors, and a variety of secondary metabolites involved in vital cellular functions [1]. In soils, sulfate is the primary source of S available to plants [2]. Plants take up sulfate primarily through the high-affinity transporters SULFATE TRANSPORTER1.1 (SULTR1;1) and SULTR1;2, with secondary contributions from SULTR2;1, while SULTR2;1 and SULTR3;5 facilitate sulfate translocation to aerial tissues. Additionally, transporters SULTR4;1 and SULTR4;2 enable the storage of S compounds in vacuoles, which serve as reservoirs for maintaining S homeostasis [3]. Within leaf tissues, sulfate is transported into chloroplasts, where ATP sulfurylase (ATPS) catalyzes its activation to form adenosine 5′-phosphosulfate (APS). APS reductase then reduces APS to sulfite, which is further reduced to sulfide by sulfite reductase. Finally, O-acetyl serine (OAS) thiol-lyase (OASTL) catalyzes the incorporation of sulfide into OAS to produce cysteine [4].

Sulfate availability in soils is a growing concern. Current environmental regulations aiming to reduce S emissions and decrease the use of S-containing pesticides have led to a marked decline in S deposition in soils. This has created scenarios where sulfate is insufficient to meet crop demands, leading to an increased use of S-containing fertilizers that can lead to detrimental long-term impacts of ecosystems [5]. Sulfate deficiency leads to stunted growth, leaf chlorosis (especially of younger leaves), and in crops, to reduced yield and nutritional value [6,7]. This last point is relevant since animals, including humans, are unable to synthesize S-containing amino acids de novo, and must obtain them from plants.

Sulfate deficiency triggers transcriptomic changes that have mainly been described in the model plant *Arabidopsis thaliana* and a few crop plants [8,9]. These studies have uncovered a diverse array of genes involved not only in S-related metabolism but also in processes linked to plant growth, hormonal regulation, defense mechanisms, and response to other nutrients [9,10,11,12,13]. SULFATE LIMITATION 1 (SLIM1), an EIN3/EIL family transcription factor (TF), has been identified as central in controlling gene expression changes in response to sulfate deficiency [14,15,16]. In addition, other TFs have been proposed as regulators of sulfate deficiency-responsive genes in *A. thaliana*, including other members of the EIN3/EIL family such as EIL1 [15] and different members of the MYB family such as MYB28 [17,18,19], among other TFs that have been reviewed in [9]. This suggests the role of complex transcriptional networks modulating the response to sulfate deficiency.

Besides transcriptional regulation, post-transcriptional control by microRNAs (miRNAs) has also been shown to impact the expression of sulfate deficiency-responsive genes. One of the most studied miRNAs in sulfate deficiency response is miR395. The miR395 family in Arabidopsis is composed of six members, of which five are upregulated by sulfate deficiency [20]. SLIM1 has been identified as a positive regulator of miR395 expression, further integrating miR395 into the broader regulatory network governing S homeostasis [21]. miR395 is able to modulate the expression of transcripts from members of the ATPS family *ATPS1*, *ATPS3,* and *ATPS4*, as well as the sulfate transporter *SULTR2;1* in Arabidopsis [20,21,22]. *ATPS* and *SULTR2* transporters have also been experimentally confirmed as targets of miR395 in other plants, including *Brassica napus* [23], *Oryza sativa* [24], *Sorghum bicolor* [25], or *Medicago truncatula* [26]. This conserved regulatory mechanism across diverse plant lineages highlights the evolutionary significance of miR395 in the sulfate deficiency response. Interestingly, an expansion of the miR395 family has been reported in some crops, such as *Oryza sativa*, whose genome encodes 25 miR395 loci [24,27], and *Solanum lycopersicum*, which presents 18 miR395 loci [28]. This suggests a complex regulatory control over sulfate metabolism by this miRNA family in these plants.

In addition to miR395, other members of miRNA families have been reported to be responsive to sulfate deficiency; however, their relevance in controlling the sulfate deficiency-responsive transcriptome as well as the physiological and metabolic reprogramming that occurs under this stress is less explored. In *A. thaliana*, miRNAs from the miR160, miR167, miR169, miR173, miR319, miR395, miR397, miR398, miR399, miR408, miR771, miR827, miR837, miR841, miR857, miR2111, miR2119, miR5632, miR5638, and miR8172 families exhibit differential expression in response to sulfate deficiency [29]. Interestingly, in *B. napus*, also belonging to the Brassicaceae family as Arabidopsis, a different set of miRNAs, including members of the miR156, miR160, miR164, miR167, miR168, and miR394 families show differential expression under sulfate limitation [23]. The difference in regulated miRNAs between those close species suggests that miRNA-mediated responses to sulfate deficiency may be species-specific. This underscores the importance of conducting further studies to unravel how miRNAs regulate sulfate deficiency across diverse plant species.

*Solanum lycopersicum* (tomato) is one of the most important vegetable crops globally, with an estimated 5.4 million ha harvested worldwide and a production of over 192 million tons in 2023 [30]. Beyond its agricultural importance, the tomato is widely recognized as a model plant for studying fruit ripening, plant metabolism, secondary metabolite biosynthesis and plant-pathogen interactions [31,32]. Sulfate deficiency has important negative effects over tomato growth, including the decreased total size of the plant, decreased shoot and root dry and fresh weight, chlorosis due to lower chlorophyll contents, diminished CO_2_ assimilation and photosynthetic activity, decreased levels of proteins, and lower fruit yield (weight and total number of fruits) [33,34,35,36,37,38,39,40,41]. Additionally, sulfate deficiency alters the homeostasis of other nutrients, such as nitrate, phosphate, calcium, magnesium, molybdenum, potassium, and iron [34,36,37,38], and causes a reduction in S-metabolites such as sulfides, cysteine, γ-glutamyl-cysteine, GSH, S-adenosyl-methionine, and an induction in serine and OAS in shoots and roots [37].

To investigate the molecular mechanisms underlying these phenotypic changes, we previously conducted a transcriptomic study on the changes induced by sulfate deficiency in tomato [40]. This analysis, which included temporal and organ-specific resolution, identified thousands of differentially expressed genes, revealing extensive transcriptome reprogramming in response to sulfate deficiency. Notably, the magnitude of this response appears to exceed that observed in classical model plants such as Arabidopsis and rice [9]. This suggests that tomato may possess distinct or specialized regulatory networks for sulfate responses, likely reflecting its physiological, developmental, and metabolic peculiarities as a fleshy-fruited plant.

Characterizing miRNAs in tomato’s post-transcriptional regulation remains an evolving field of study. Current studies of tomato have addressed the roles of miRNAs in various biological processes, including fruit ripening [42,43] and abiotic [44] and biotic stress responses [28,44,45]. However, the full extent of miRNA-mediated regulation in the sulfate deficiency response remains largely unexplored in tomato.

In this work, we performed an organ- and time-specific sequencing of the sRNA fraction of the transcriptome to comprehensively characterize the sulfate deficiency-responsive miRNAome. We further integrate these data, incorporating evidence from degradome studies and transcriptomic datasets, to identify putative miRNA targets and reconstruct miRNA-centered gene regulatory networks underlying the extensive transcriptome reprogramming induced by sulfate deficiency in leaves and roots. Finally, we compare these networks to those described in *Arabidopsis thaliana*, revealing both conserved and species-specific aspects of miRNA-mediated regulation of sulfate deficiency-responsive gene expression.

## 2. Results

### 2.1. Annotation of miRNA Genes in the SL4.0 Genome

Current information on tomato miRNA genes has been generated using different genome assemblies, including SL2.5 [27,46,47] and SL3.0 [28,48,49]. However, the SL4.0 genome assembly, produced by the International Tomato Genome Sequencing Project and maintained at the Sol Genomics database [50], has become a current reference and is widely used in genomic and comparative genomics studies of tomato [51,52,53,54,55,56].

To generate an updated annotation of miRNA genes mapped to the SL4.0 genome, we integrated data of hairpin and mature miRNA sequences from three main sources: (i) the work by Cardoso et al. [48], (ii) the miRBase database [27], and (iii) the Plant small RNA genes database [46]. This combined dataset resulted in 472 hairpin precursor sequences. These precursors were aligned to the SL4.0 genome using BLAST 2.16.0 to determine their genomic coordinates (Table S1). For overlapping miRNA loci identified by multiple sources, we assigned locus identifiers following the priority order: miRBase, Cardoso et al. [48] and the Plant small RNA genes database. After removing redundant hairpin precursors, we retained a total of 347 unique miRNA sequences (Table S2). These sequences comprise miRNAs from 111 distinct miRNA families, along with 82 sequences from the Plant small RNA genes database that fulfilled criteria for miRNA annotation, but were not assigned to a known family, potentially representing tomato-specific miRNAs. This updated annotation was subsequently used to identify sulfate deficiency-responsive miRNAs and is provided in GFF format to facilitate its use by the research community (Table S3).

### 2.2. Tomato miRNAs Are Regulated in an Organ- and Time-Dependent Manner in Response to Sulfate Deficiency

Sulfate deficiency negatively impacts plant growth and development, inducing significant transcriptomic changes in tomato leaves and roots [40]. To understand the role of miRNAs in the post-transcriptional control of gene expression in response to sulfate deficiency, we grew tomato plants under sulfate-sufficient and sulfate-deficient conditions for 3 and 4 weeks. These time points were selected as we found they induce massive changes in gene expression in tomato [40]. As a validation step, we evaluated plant growth parameters, sulfate contents, and expression of sulfate deficiency markers, replicating the experimental conditions from our previous transcriptomic analysis [40]. We found that sulfate deficiency caused a reduction in leaf and root growth at both time points (Figure S1A,B), as well as a reduction in sulfate contents (Figure S1C), and an induction of the *APS reductase* (*APR*) (*Solyc02g080640*), *SULTR* (*Solyc04g072760*), and *EIL3* (*Solyc01g006650*) genes, as previously reported [40] (Figure S1D–F).

We extracted total RNA from roots and leaves under both conditions and sequenced the sRNA component. We aligned sRNA-seq data to the SL4.0 tomato reference genome using ShortStack, a de novo sRNA loci discovery tool [57,58]. While alignment rates were higher and more uniform in leaves than in roots, the observed values are well within the range reported for sRNA libraries from tomato and other plant species in both organs [46] (Table S4).

**Figure 1:**
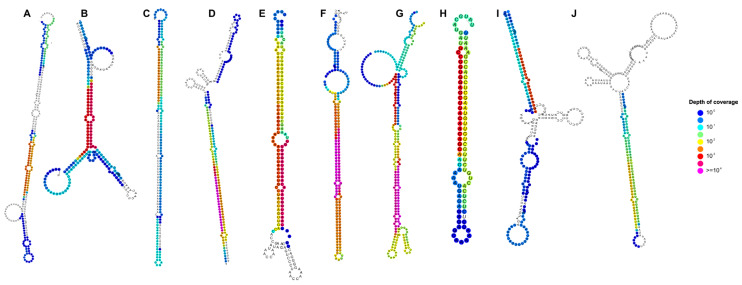
ShortStack analysis of sRNA species from leaves and roots of plants exposed to control and sulfate deficiency conditions identifies ten novel miRNAs. *Solanum lycopersicum* cv. Moneymaker seeds were germinated and grown in hydroponic medium containing modified basal 0.5 X Murashige and Skoog salts containing K_2_SO_4_ as sulfate source (control condition) or KCl (treatment, sulfate deficiency condition). Predicted hairpins from sRNA clusters passing all ShortStack tests for miRNA genes, and not annotated as a known miRNA, were extracted and their secondary structures were predicted using StrucVis 0.9 (https://github.com/MikeAxtell/strucVis, accessed on 10 October 2024). The color gradient shows the depth of alignment coverage. (**A**): sly-b4.0r1-14993_MIRNA; (**B**): sly-b4.0r1-14505_MIRNA; (**C**): sly-b4.0r1-21667_MIRNA; (**D**): sly-b4.0r1-25391_MIRNA; (**E**): sly-b4.0r1-59201_MIRNA; (**F**): sly-b4.0r1-60115_MIRNA; (**G**): sly-b4.0r1-66932_MIRNA; (**H**): sly-b4.0r1-83697_MIRNA; (**I**): sly-b4.0r1-96827_MIRNA; and (**J**): sly-b4.0r1-97328_MIRNA.

ShortStack identifies miRNA loci based on the alignment patterns of reads to a reference genome. For valid miRNA loci, the alignment should produce a discrete pattern to a single strand, clearly distinguishing a major species (mature miRNA) from the minor species (complementary miRNA), separated by a short distance. Moreover, ShortStack requires that the major species coincide with the typical sizes for DCR-produced miRNAs in plants (21–22 nt) [57,58]. ShortStack analyses resulted in the identification of 94,723 sRNA clusters with Dicer call (size of the most abundant sRNA) of 20–24 across all samples (Table S5). We found sRNA clusters corresponding to 259 miRNA genes with nomenclature assigned according to our SL4.0 annotation. Interestingly, ShortStack identified 10 loci that passed all criteria for miRNA annotation (classified as “Y” by ShortStack’s MIRNA analysis) [57]. For these new miRNAs, we evaluated the secondary structure of the predicted precursor, finding a typical stem-loop structure, supporting their potential as bona fide miRNAs (Figure 1). We assigned these loci with the identifier “sly-b4.0-sRNAcluster#_MIRNA” and added them to the miRNA annotation (Tables S2 and S3).

**Figure 2:**
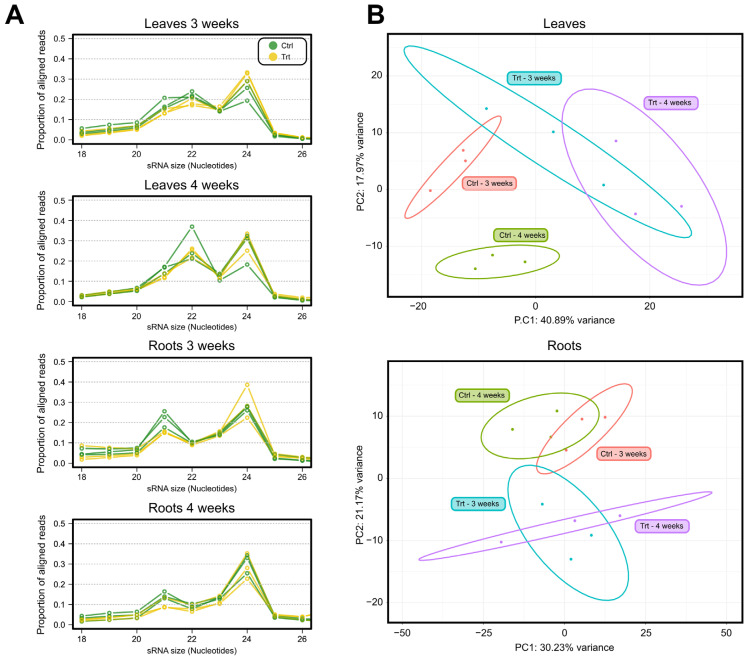
Size distribution and expression patterns of sRNAs in response to sulfate deficiency. *Solanum lycopersicum* cv. Moneymaker seeds were germinated and grown in hydroponic medium containing modified basal 0.5 X Murashige and Skoog salts containing K_2_SO_4_ as sulfate source (Ctrl and control condition) or KCl (Trt, treatment, and sulfate deficiency condition). (**A**) Size distribution of sRNA species across organs and time points. (**B**) Principal Component Analysis (PCA) of the expression of the sRNA clusters of 20–24 nt across conditions. PCA was conducted using the pcaExplorer 3.2.0 R package with log_2_-transformed, normalized expression data. Ellipses represent the 95% confidence interval based on three independent experiments. Replicates of the same experiment are represented with identical colors.

Consistent with the size distribution of sRNA reads reported in tomato and other plants [59,60,61,62], we observed typical sizes of plant sRNAs in our libraries, ranging between 20 and 24 nucleotides (Figure 2A). We found similar distributions of reads between sulfate sufficiency and deficiency conditions in both organs (Figure 2A). This finding indicates that sulfate deficiency does not impact the size distribution of expressed sRNA species. We observed a shift in the distribution of sRNA reads across organs, with leaves displaying a higher proportion of 22 nt species, whereas roots exhibited a sharp peak at 21 nt. This pattern aligns with findings from previous studies in tomato leaves [63,64,65].

We performed a Principal Component Analysis (PCA) to explore variations in sRNA expression profiles across samples (Figure 2B). For this analysis, we focused on sRNA counts within the 20–24 nt size range, which aligns with the typical size distribution of plant sRNAs. The PCA separates sulfate sufficiency from sulfate deficiency in leaves and roots. In leaves, we observed a further separation of control samples based on time, distinguishing the 3-week and 4-week times. In contrast, root samples from control conditions clustered closely together (Figure 2B). These results suggest that aerial tissues exhibit a time-dependent expression of miRNAs that is dependent on the sulfate content, which is consistent with previous findings for the mRNA component of the transcriptome [40].

To identify sulfate deficiency-responsive miRNAs in roots and leaves, we analyzed read counts mapped to our miRNA annotation and analyzed differential expression with the DESeq2 R package. We identified 40 miRNAs that are differentially expressed (DE) in at least one comparison between sulfate deficiency and sufficiency conditions (11.2% of total annotated miRNAs) corresponding to 23 different families (20.7% of total families). These 40 miRNAs include 3 miRNAs from The Plant small RNA genes database not classified into a family (Lunardon2020_sly-b2.5r1-49254_MIRNA, Lunardon2020_sly-b2.5r1-31088_MIRNA and Lunardon2020_sly-b2.5r1-147852_MIRNA), as well as 2 of the new miRNAs identified in this work (sly-b4.0r1-14505_MIRNA and sly-b4.0r1-14493_MIRNA) (Table 1 and Table S6).

**Figure 3:**
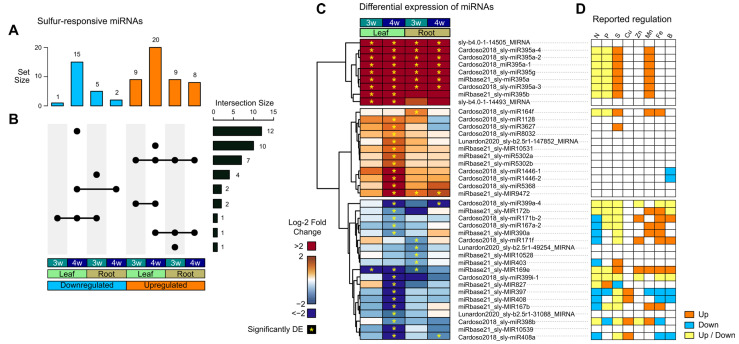
miRNAs show organ- and time-specific responses to sulfate deficiency in tomato. *Solanum lycopersicum* cv. Moneymaker seeds were germinated and grown in hydroponic medium containing modified basal 0.5 X Murashige and Skoog salts containing K_2_SO_4_ as a sulfate source (control condition) or KCl (treatment and sulfate deficiency conditions). Three biological replicates, each consisting of a pool of five plants per condition, were used. Differentially expressed miRNAs were determined by DESeq2 analysis comparing the treatment versus control condition. (**A**) Bar plot representing the number of unique differentially expressed (DE) miRNAs in each sample, highlighting temporal and organ-specific dynamics. (**B**) Overlap analysis of DE miRNAs between different samples, showing shared and unique responses. (**C**) Hierarchical clustering analysis (HCA) of expression of sulfate-responsive miRNAs. Hierarchical clustering was performed with hclust in R, considering the complete linkage method and Euclidean distance. Colors represent the log_2_ fold change in the expression of all DE miRNAs across the samples. Yellow asterisks in the center of each cell denote significant changes in miRNA expression determined by DESeq2 (Wald test, *p* < 0.05 and log_2_ fold change > 0.5). (**D**) Information on miRNA responses to S and other nutrient deficiencies in other plants according to [66] is included in the right-hand cells, showing whether the miRNA is upregulated (Up, orange), downregulated (Down, blue), or upregulated or downregulated depending on the plant/treatment (Up/Down, yellow) [66].

We found that the duration of sulfate deficiency impacted miRNAs in a different manner, depending on the organ. While leaves and roots presented a similar number of DE miRNAs at week 3 (10 DE miRNAs in leaves and 14 DE miRNAs in roots), prolonged sulfate deficiency principally affected the DE of miRNAs in leaves, with 35 DE miRNAs at week four (15 downregulated and 20 upregulated) compared to only 10 DE miRNAs at week four in roots (2 downregulated and 8 upregulated) (Figure 3A). A more detailed analysis showed that the majority of DE miRNAs exhibited a time- and organ- dependent response to sulfate deficiency, with 27 miRNAs (67.5% of DE miRNAs) responding exclusively in a single organ at a single time point, indicating a transient and organ-specific response. Despite this trend, a relevant subset of miRNAs (11 miRNAs, 27.5%) responded in both leaves and roots. Interestingly, the expression changes were consistent across organs, being either upregulated or downregulated in both leaves and roots (Figure 3B).

Hierarchical clustering analysis of DE miRNAs showed three main clusters of expression (Figure 3C). The first cluster includes miRNAs that are consistently and highly induced by sulfate (log_2_ fold-change > 3) in roots and leaves. This cluster includes seven members of the miR395 family, confirming a conserved and central role of miR395 in the tomato sulfate deficiency response, as described in other plants. Additionally, this cluster contains two new miRNAs, identified by ShortStack, sly-b4.0r1-14493, and sly-b4.0r1-14505, that were strongly induced by sulfate deficiency. sly-b4.0r1-14493 showed induction in leaves at 3 and 4 weeks of sulfur deficiency, while sly-b4.0r1-14505 was upregulated in both organs at both time points (Figure 3C). The second cluster includes 12 miRNAs from 10 distinct families that are predominantly induced in leaves after four weeks of sulfate deficiency, with only a couple of them showing significant upregulation in roots. Little is known about the function of these miRNAs in plants, since most of them correspond to species-/lineage-specific miRNAs [47] (Figure 3C).

Concerning miRNAs with known functional roles in plants, Cluster 2 contains miRNAs related to organ growth and stress responses, including one member of the conserved miR164 family [67,68,69,70,71,72,73], miR1128 [74], and the Solanaceae-specific miRNAs miR3627 [75,76] and miR9472 [77]. The third cluster includes miRNAs that are repressed in leaves and/or roots, particularly at 4 weeks in leaves and at 3 weeks in roots. This cluster includes an important proportion of conserved miRNAs with well-established roles in plant growth, development, stress, and nutrient responses, including members of the miR167, miR171, miR172, miR390, miR397, miR398, miR399, miR403, and miR827 families [28,44,78].

Interestingly, the response to sulfate deficiency of miRNAs from Clusters 1 and 3 in other plants is consistent only for a few miRNAs (miR395, miR164, miR3627 and miR827), with the other miRNAs appearing either induced or repressed depending on the specific study (Figure 3C). Moreover, most of the miRNAs in Cluster 2 have not been reported as sulfate-responsive in other studies. Most of the conserved miRNAs in Clusters 1 and 3 are also responsive to N or phosphate deficiency, while some of them also respond to deficiencies of Cu, Zn, Mn, Fe, or B in different plant species including *Arabidopsis thaliana*, *Zea mays*, *Sorghum bicolor*, *Oryza sativa*, *Taxus chinensis*, *Citrus chinensis*, *Arachis hypogaea*, *Phaseolous vulgaris*, *Triticum aestivum*, or *Brassica juncaea* [66] (Figure 3C).

### 2.3. Analysis of Regulatory Elements in miRNA Promoters Shows Enrichment of TF Families Involved in Plant Responses to Stress

In order to define potential transcriptional regulators of DE miRNAs, we extracted upstream promoter regions from each miRNA precursor and conducted a cis-motif enrichment analysis using the Analysis of Motif Enrichment (AME) tool [79] in the MEME suite [80] (Table S7). The transcriptional start site of miRNAs was defined as the start of the reported miRNA precursor in miRBase, the work of Cardoso et al. [48], or the Plant small RNA genes database, in line with our annotation criteria. In the absence of experimental data defining miRNA gene promoters in tomato, we defined a distance of 3000 bp upstream of the TSS, as this distance has been reported to include most open chromatin sites in tomato [81]. Among the enriched motifs, several TF families involved in organ development and/or stress were highlighted, notably DNA-binding with one finger (DOF), Apetala2/Ethylene response factor (AP2/ERF), and NAM, ATAF1/2, CUC2 (NAC), with members predicted to regulate miRNA expression in both roots and leaves. Within the DOF family, we identified motifs matching Arabidopsis TFs such as CDF3, CDF5, COG1, OBP1, OBP3, and OBP4. In the AP2/ERF family, overrepresented motifs correspond to Cytokinin response factors (CRFs), as well as ERFs and Dehydration-responsive element binding (DREB) A-2 subgroup members (DREB2-types). NAC motifs included those for Cup shaped cotyledon (CUC1-3), and factors such as NAC2, NTM1, and NTM2. Other less-represented families included B3, bHLH, bZIP, C2H2, GATA, HD-ZIP, MYB, WRKY, and TCP, all of which harbor members involved in hormonal regulation, development, and responses to environmental stimuli [82,83]. Importantly, we detected enrichment of the consensus binding site for SLIM1/EIN3 in DE miRNA promoters from roots and leaves (Table S7). This suggests that a tomato homolog of Arabidopsis SLIM1 may play a conserved regulatory role in controlling sulfate-responsive miRNAs.

### 2.4. Organ-Specific Targets of Sulfate-Responsive miRNAs Reveal Distinct Regulatory Programs in Leaves and Roots

To explore the impact of DE miRNAs in regulating sulfate deficiency-responsive genes, we predicted targets for all DE miRNAs using publicly available Degradome-seq libraries from diverse tomato organs and conditions (Table S8). Target genes were retained when Degradome-seq data provided evidence of miRNA-guided cleavage, and results were filtered to include only mRNAs DE under sulfate deficiency in leaves or roots at 3 and/or 4 weeks, based on a re-analysis of the data from Canales et al. [40] using the SL4.0 genome and ITAG4.0 annotation (Table S9). This analysis identified experimentally validated targets (at least from one Degradome-seq experiment) for 16 of the 40 DE miRNAs, 11 in leaves, 2 in roots and 3 shared, spanning 10 known miRNA families, one unclassified miRNA and one novel miRNA (Tables S10 and S11). We found 34 miRNA–target interactions in total, with the vast majority (29 of 34) occurring in leaves, suggesting a more extensive miRNA-mediated regulatory program in aerial tissues during sulfate deficiency. Interestingly, a substantial fraction of miRNA–target pairs (19 of 34) exhibited the same directional response to sulfate, with both the miRNA and its target being upregulated or downregulated. This includes classic miR395–target pairs such as ATP Sulfurylase (*Solyc09g082860*) and a sulfate transporter-like gene (*Solyc04g054730*), which were induced alongside miR395 in both leaves and roots. Although seemingly contradictory to the canonical repressive role of miRNAs, this phenomenon has been previously reported in Arabidopsis for miR395 and its targets (e.g., *SULTR2;1* or *ATPS3*), and has been attributed to non-overlapping expression domains between miRNAs and their targets at the cellular level [20]. Given this, we included both positively and negatively correlated pairs in our analysis (Table 2 and Table S11). Beyond its canonical sulfur-related targets, miR395 was also linked to regulation of broader metabolic processes in leaves, targeting the transcripts of dihydrolipoamide dehydrogenase (Solyc05g05330), the thylakoid protein proton gradient regulation 5 (PGR5)-like protein 1A (Solyc08g080050), and a chloride channel (Solyc10g005690) (Figure S2, Table 2 and Table S11).

Among other differentially expressed miRNAs, miR167, typically known to regulate *Auxin Response Factors*, was found to target the *SlWDR156 WD40 repeat protein transcript* (*Solyc07g008860*). miR172 targeted *AP2-like ethylene-responsive transcription factor* (*Solyc04g049800*) and *ZAT11*, a zinc finger protein. In line with its established role in phosphate homeostasis, miR827 targeted the *PHT5;32 SPX domain containing protein* (*Solyc08g007800*). Additionally, miR1446 targeted a *2-alkenal reductase (NADP(+)-dependent)-like protein* (*Solyc09g091700*).

Less characterized differentially expressed miRNAs also revealed interesting targets. miR5638 targeted five DE transcripts in leaves, including *Constitutive photomorphogenic 1 (COP1)-interacting protein 4* (*Solyc05g014130*), a *nucleotide diphospho sugar transferase* (*Solyc06g066800*), a *2-alkenal reductase* (*Solyc07g045080*), and enzymes associated with redox metabolism. miR10539 regulated the transcript of the *NF-YB3c* transcription factor (*Solyc12g006120*), *stress-related glycine-rich protein* (*Solyc02g06340*), and a *mitochondrial carnitine/acylcarnitine carrier-like protein* (*Solyc10g079200*). The Solanaceae-specific miR5302 targeted two *serine carboxypeptidases* (*Solyc04g077640* and *Solyc04g079040*) and an *acyl-CoA N-acyltransferase*. We were also able to assign an *alpha/beta hydrolase* (*Solyc09g009540*) as target to the novel miRNA sly-b4.0r1-14493_MIRNA in leaves, further supporting its assignment as a bona fide miRNA.

In contrast, root miRNA–target pairs were more limited, with only five interactions identified. Three corresponded to canonical miR395 targets, while miR10528 was found to target a *tRNA threonylcarbamoyladenosine dehydratase* (*Solyc05g051230*), and Lunardon2020_sly-b2.5r1-49254_MIRNA a *zinc transporter* (*Solyc07g065380*).

### 2.5. Cross-Species Comparison Reveals Broader miRNA Regulatory Functions in Tomato

**Figure 4:**
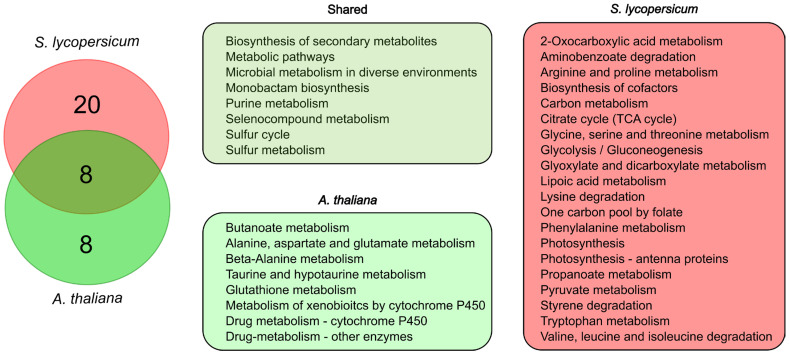
Comparative analysis of metabolic processes controlled by DE miRNAs in *Arabidopsis thaliana* and *Solanum lycopersicum*. DE miRNA targets were analyzed using BlastKOALA [84] to determine their participation in KEGG pathways. For *Arabidopsis thaliana*, DE miRNAs were obtained from [85]. Targets were assigned based on information from TarDB [86], filtering for sulfate-responsive transcripts obtained from [29].

Finally, to evaluate the conservation and divergence of metabolic processes regulated by miRNAs under sulfate deficiency, we compared the KEGG pathway annotations of Degradome-seq-validated targets of sulfate-responsive miRNAs in *Arabidopsis thaliana* and *Solanum lycopersicum* using BlastKOALA [84]. DE miRNAs in *Arabidopsis thaliana* were obtained from [85] while evidence of targeting for miRNAs was obtained from TarDB (http://www.biosequencing.cn/TarDB/, accessed on 16 June 2025) [86], considering information based on Degradome-seq. Arabidopsis targets were filtered based on sulfate deficiency-responsive genes obtained from the study by [28]. A total of 70 miRNA–target pairs were found for Arabidopsis, including pairs consisting of members of the miR167, miR169, miR172, miR2111, miR390, miR395, miR408, miR827, and miR857 families (Table S12). Eight KEGG pathways were shared between the two species (Figure 4), including sulfur cycle and metabolism, selenocompound and purine metabolism and pathways associated with biosynthesis of secondary metabolites. In contrast, a substantial number of pathways were species-specific. Tomato miRNA targets were involved in twenty unique KEGG categories, which included several central metabolic pathways such as glycolysis/gluconeogenesis, the tricarboxylic acid (TCA) cycle, glyoxylate and pyruvate metabolism, amino acid metabolism and degradation pathways, as well as photosynthesis. On the other hand, Arabidopsis miRNA targets were involved in eight exclusive pathways, including glutathione metabolism, detoxification-related pathways, butanoate metabolism, and metabolism of the amino acids alanine, aspartate, and glutamate (Figure 4).

## 3. Discussion

### 3.1. An Updated Annotation of miRNAs on the SL4.0 Genome

miRNAs are crucial molecules that play a central role in regulating organism growth and development in response to both external and internal signals. Identifying and annotating miRNAs is a critical first step toward understanding their specific biological functions. Over the past two decades, significant efforts have been made to generate sRNA sequencing data, annotation tools, and databases to compile information on miRNA genes. While several databases currently house miRNA gene annotations, these annotations are inconsistently reported across different versions of the tomato reference genome. This inconsistency poses challenges when working with newer and updated genome assemblies. To address this issue, we focused on creating a comprehensive miRNA annotation for the tomato SL4.0 genome, the latest version available in the Sol Genomics Network database. Through our analysis, we annotated a total of 357 miRNA loci, which is a higher number than previously described (e.g., 226 miRNA genes found in [48], 194 in [47], 112 in miRBase or 173 in [46]), but lower than the number in a recently reported tomato *MIR* atlas (538 genes, [28]). Notably, an important part of these miRNAs were annotated as miRNAs or near-miRNAs (passing all tests, except the presence of a miRNA*) in [46], but are not associated with a specific miRNA family, suggesting they can represent species-specific miRNAs. Consistently, a remarkable percentage of tomato miRNAs have been described as being species-specific [28], indicating a recent evolutionary history and likely a role in fine tuning of lineage-specific processes. An additional source of miRNAs not previously annotated in known families arose from the de novo annotation of sRNA loci performed by ShortStack during the analysis of the sulfate deficiency libraries. Through this approach, we identified 10 novel miRNAs, indicating that the current tomato miRNA repertoire remains incomplete. This contrasts with *Arabidopsis thaliana*, where a broad range of conditions and organs have been profiled. In tomato, most sRNA studies have focused on fruit development or responses to pathogen infection in leaves or roots [28]. Our findings highlight the need for systematic exploration of sRNA expression across a wider range of tissues, developmental stages, and stress conditions to fully uncover the diversity and regulatory potential of miRNAs in tomato.

### 3.2. miRNA Responses to Sulfate Deficiency Are Organ- and Time-Specific and Integrate Crosstalks with Other Nutrients, Development, and Stress Responses

Previous studies of sulfate deficiency-responsive miRNAs in plants have focused on seedlings at a single developmental stage [29,85]. In contrast, our study is the first to provide a comprehensive temporal and organ-specific perspective of miRNA responses to sulfate deficiency, enabling a deeper understanding of the distinct biological processes regulated by miRNAs in roots and leaves under this stress. Interestingly, we observed strong organ- and time-dependent patterns of miRNA regulation. Among the 40 DE miRNAs, only 11 responded in both organs (3 downregulated and 8 upregulated), while 29 (18 downregulated and 11 upregulated) displayed time-specific expression changes. The majority of DE miRNAs were found in leaves (35 miRNAs), with most of them showing late induction (25 were DE only at four weeks of sulfate deficiency). In roots, we found fewer DE miRNAs (16 in total), but their responses tended to occur earlier (at three weeks) and were more evenly distributed between time-dependent (8 miRNAs) and time-independent (8 miRNAs) patterns. This regulatory behavior closely matches transcriptomic responses to sulfate deficiency, where root gene expression changes precede those in leaves [40]. Indeed, after two weeks of sulfate starvation, transcriptomic changes were detectable only in roots [40], showing their primary role as the initial site of nutrient sensing and signaling.

While transcriptomic responses to sulfate deficiency have been investigated across different plant species [9], the current scenario for miRNA-mediated regulation under these conditions remains comparatively less explored. To date, sulfate deficiency-responsive miRNAs have been reported in only a limited number of plant species, including *Arabidopsis thaliana* [29,85], *Brassica napus* [23], and *Brassica juncaea* [87]; however, only the report of Liang et al., 2015 [85], on Arabidopsis is based on the high-throughput sequencing of the sRNA component of the transcriptome. We found that the number of sulfate deficiency-responsive miRNAs in tomato (21 induced and 19 repressed miRNAs) is similar to those reported in *Arabidopsis thaliana* (18 induced and 19 repressed miRNAs) [85]. However, only members of 10 conserved families were responsive in both species, pointing to the existence of a conserved miRNA regulatory core alongside substantial species-specific regulation.

Most of these miRNAs include species whose canonical targets are involved in the homeostasis of nutrients. These include miR399, targeting *ubiquitin conjugase E2* and miR827, targeting *ubiquitin E3 ligase NLA*, both involved in phosphate uptake and translocation. While these miRNAs are repressed by sulfate deficiency in tomato and also in Arabidopsis [85], they are induced by phosphate deficiency [88,89,90]. A similar case has been shown for miR395, where sulfate deficiency induces while phosphate deficiency represses its expression [88,89,90]. This antagonistic regulation of miRNAs might arise from a coordinated adaptation to balance nutrient acquisition and allocation to control the biosynthesis of different compounds, such as increasing sulfolipid biosynthesis when phosphate is low and increasing phospholipid biosynthesis when sulfate is low. This miRNA-mediated mechanism might complement other regulatory mechanisms of nutrient crosstalk such as direct regulation of sulfate transporters by the Phosphate starvation response 1 (PHR1) TF, a protein that regulates the response to phosphate deficiency [91]. Other common regulated miRNAs included those with copper homeostasis-related targets, miR397 and miR408, targeting cuproproteins such as laccases and miR398, targeting copper superoxide dismutases. These miRNAs are downregulated by sulfate deficiency, and upregulated by copper deficiency via the SPL7 transcription factor [92,93]. Under copper deficiency, SPL7-mediated control of these miRNAs downregulates non-essential copper proteins to conserve copper for essential enzymes like plastocyanin, involved in photosynthesis. Under sulfate deficiency, a downregulation of these miRNAs might translate to a need for managing reactive oxygen species, due to the decrease in glutathione levels, depending more on Cu-containing enzymes for detoxification. miR169, a miRNA involved in N starvation response, is also repressed by sulfate deficiency in Arabidopsis and tomato. Similarly, this miRNA is repressed by N starvation, leading to an induction of its NFYA targets in roots and shoots. miR169 overexpression leads to a repression of nitrate transporters NRT1.1 and NRT2.1, suggesting the involvement of miR169 in N uptake [94]. Also, miR169 is involved in stress responses by controlling the expression of its target NFYA5, leading to the induction of genes involved in managing oxidative stress and leading to drought and salt tolerance [95,96]. Thus, sulfate deficiency repression of this miRNA might serve to counteract the effects of oxidative stress but also to control N uptake, given the need to coordinate S and N metabolites for amino acid biosynthesis. A recent work showed that defects in S signaling caused by mutation of the *Chlorophyll A/B-binding (CAB) overexpression 2* (*COE2*) gene severely disrupt N acquisition and assimilation, highlighting the strong interdependence of S and N pathways [13]. Interestingly, this regulation also involved miRNA-mediated processes, where miR396a and miR396b were upregulated in *coe2* leaves under sulfate deficiency, leading to the repression of their targets, members of the Growth regulating factor protein family [13].

In addition, our analysis highlighted several known miRNAs linked to developmental and stress response pathways. miR164, by targeting members of the *NAM, ATAF1/2, and CUC2* (*NAC*) TFs, may integrate developmental programs such as organ boundary specification and leaf or root growth with stress adaptation, suggesting a regulatory trade-off between growth and stress tolerance. miR1128, previously described in wheat as regulating a *lecithin-cholesterol acyltransferase* (*LCAT*) family [74], could similarly modulate lipid metabolism under sulfate stress in tomato, a pathway strongly affected by sulfate deficiency in this organism [40]. Interestingly, miR3627 from the miR4376 superfamily targets *Ca*^2+^*-ATPase10* homologs during tomato reproductive growth [75,76], indicating that sulfate deficiency may influence reproductive processes and ultimately yield-related traits. In addition, sulfate-dependent regulation of miR9472 might impact salicylic acid (SA) biosynthesis and pathogen defense, since this miRNA has been reported to target *Sar deficient 1* (*SARD1*), a central regulator of SA biosynthesis underlying the resistance to *Cladosporium fulvum* in tomato [77].

Concordant with the idea that sulfate deficiency responses intersect with broader networks of nutrient availability, development, and stress adaptation, our motif enrichment analysis suggests that sulfate-responsive miRNAs may be regulated by a diverse array of TFs. For instance, CDFs and OBPs, which are classically linked to photosynthesis, light signaling, flowering time, and cell expansion [97,98,99,100,101,102,103,104,105,106,107], may connect energy and developmental programs with sulfate status. Similarly, regulation by CRFs, ERFs, and DREBs might connect growth and abiotic stress [108,109] to nutrient deficiency signaling. The detection of CUC motifs, as well as NAC and NTM motifs, further suggests a link to root growth regulation and germination processes [110,111,112,113]. Finally, enrichment of additional motifs from families such as bZIP, MYB, and WRKY points to the integration of hormonal and environmental cues into the transcriptional control of sulfate-dependent miRNA responses [82,83].

A notable finding was the overrepresentation of the SLIM1/EIL3 motif in sulfate-responsive miRNA promoters. In Arabidopsis, the SLIM1 TF is a well-established central controller of sulfate uptake- and metabolism-related genes, as well as of other sulfate deficiency-responsive genes, including members of the miR395 family [14,21]. In tomato, the SLIM1 homolog *Sl*EIL3 (Solyc01g006650) has been proposed to play a similar role in the sulfate deficiency response [9,40]. However, experimental validation of its function in tomato is still lacking, and further studies are required to confirm its role in this process.

Taken together, these findings indicate that sulfate-responsive miRNAs act within a multilayered regulatory framework where nutrient status is closely coordinated with developmental and stress-related pathways.

### 3.3. Expanded Functional Roles and Targets of Sulfate-Responsive miRNAs in Tomato

Although members of multiple miRNA families were DE by sulfate deficiency, we identified DE targets with experimental evidence of cleavage for only 16 of the 40 DE miRNAs. While these libraries encompass diverse organs and experimental conditions, it is likely that additional miRNA–target interactions occur specifically under sulfate-deficient conditions and remain undetected in the current dataset. Thus, the full landscape of miRNA-mediated regulation in this context may be incomplete. An alternative strategy used in other tomato miRNA studies [65,114,115,116] involves bioinformatic prediction of targets based on sequence complementarity and thermodynamic properties. However, such approaches often generate a high rate of false positives due to their reliance on in silico parameters and lack of tissue or condition-specific validation. Ideally, Degradome libraries or equivalent approaches performed under sulfate deficiency conditions would provide a more accurate and complete representation of the complete miRNA–target network. Despite these limitations, our analysis recovered several well-established miRNA–target pairs that are likely relevant in the context of sulfate deficiency. These include miR395 targeting *ATP sulfurylase* and *Sulfate transporter-like* genes, miR172 targeting an *AP2-like* transcription factor, miR398 targeting a *Superoxide dismutase*, and miR827 targeting an *SPX domain-containing protein*. In addition to these canonical interactions, our results also revealed novel miRNA–target pairs with potential functional significance during sulfate deficiency, pointing to a broader and more dynamic regulatory role for miRNAs under this stress condition. Interestingly, many of these interactions involved positively co-regulated miRNAs and targets. While plant miRNAs canonically reduce target RNA levels through cleavage, such positive co-regulation can arise through different mechanisms. For instance, in the case of miR395, sulfate deficiency has been shown to induce both miR395 and their targets, but in different cell types of the root [20]. In *Zea mays*, miR308a and its target *3-oxo-Delta(45)-steroid 5-betareductase* (*VEP1*) show inverse expression trends in mature zones of the root, but a concordant expression in meristematic zones under chilling stress [117]. Thus, apparent co-induction at the bulk tissue level may reflect tissue-, developmental-, or cell-type-specific regulation, allowing both transcripts to accumulate when measured across whole organs. Alternatively, complex regulatory loops might simultaneously promote the expression of both the miRNA and its target. This has been reported in nitrate responses, where an incoherent feed-forward loop controls miR393 and its target, the auxin receptor *Auxin signaling F-box 3* (*AFB3*) in Arabidopsis [118]. Nitrate initially induces *AFB3*, while N metabolites produced downstream nitrate reduction and assimilation induce miR393 to repress *AFB3* expression in roots during prolonged exposure to this nutrient. Similar regulatory logic may underlie some of the positive miRNA–target correlations observed here.

For miR395, the most extensively studied miRNA in the context of sulfate deficiency in plants, we identified three downregulated targets in leaves that are consistent with its upregulation under sulfate-limited conditions. One of these targets encodes a *chloride channel*, which aligns with recent findings suggesting that chloride channels in tomato are regulated by several miRNA families, including miR395 [119]. Given that chloride channels contribute to anion homeostasis, cell volume regulation, and responses to abiotic stress [120], their downregulation may help prevent ionic imbalance and restrict energy-costly non-essential ion fluxes, especially since chloride transport is tightly coupled to proton gradients and maintaining membrane potential. The other two downregulated targets, *dihydrolipoamide dehydrogenase* and *PGR5-like protein 1A* are both associated with photosynthesis. This finding is consistent with known effects of sulfate deficiency on photosynthesis, which is impaired due to reduced synthesis of essential S-containing factors (e.g., plastocyanin and Fe-S clusters in ferredoxin) and decreased antioxidant capacity (e.g., glutathione and thioredoxin). Although previous studies have attributed miR395-associated phenotypes in plant growth, stress responses, and pathogen resistance primarily to its canonical targets, our results suggest that non-canonical targets, such as those identified here, could also contribute to miR395 mediated regulation. Supporting this, the transcripts for WRKY TFs involved in leaf spot resistance have been described as non-canonical targets of miR395 in apple [121].

Beyond miR395, our results reveal that sulfate deficiency-responsive miRNAs participate in regulating a wide array of biological processes. Among the downregulated targets, many are involved in core cellular functions such as redox balance and detoxification (e.g., *NADPH-dependent reductases*), energy and primary metabolism (e.g., TCA cycle enzymes, photorespiration, cyclic electron flow, and lipid metabolism), nutrient transport and homeostasis, and protein metabolism, pointing toward a strategy to reallocate resources and maintain metabolic balance under sulfate deficiency. Conversely, upregulated targets are involved in processes related to protein degradation and remodeling (e.g., proteases and proteasome components), along with redox-related enzymes such as *superoxide dismutase*. This pattern suggests an enhanced protein recycling mechanism, likely to recover S-containing amino acids, and an increase in antioxidant activity to counteract elevated levels of reactive oxygen species caused by sulfate deficiency.

Importantly, despite limited overlap in specific targets, the coordinated regulation of functionally interconnected pathways implies a convergence of miRNA activity toward a common adaptive goal. This supports a model in which miRNAs orchestrate finely tuned trade-offs between growth, defense, and nutrient use efficiency during sulfate stress. The discovery of tomato-specific miRNAs, the expanded target space for known miRNAs, and the broader functional diversity of their targets compared to Arabidopsis further highlights the evolutionary diversification of miRNA-mediated regulation. Taken together, these findings position miRNAs as pivotal and context-sensitive regulators that integrate developmental, metabolic, and environmental signals in the sulfate deficiency response.

## 4. Materials and Methods

### 4.1. miRNA Annotation in the SL4.0 Genome Assembly

miRNA hairpins and mature miRNA sequences for tomato were obtained from miRBase version 22.1 [27], the Plant small RNA genes database [46], and the work by Cardoso et al. [48]. To generate an updated annotation of miRNAs for tomato, hairpin sequences were aligned to the tomato SL4.0 genome using BLAST [122] with alignment parameters set to an identity threshold of >95% and an e-value < 0.01. Genomic coordinates of aligned sequences were retrieved, and miRNA gene names were assigned based on their designations in the original publications. For redundant miRNA genes, naming priority was given to their miRBase names, followed by names given in Cardoso et al. [48], and then in the Plant small RNA genes database [46].

### 4.2. Plant Material and Growth Conditions

Seeds of *Solanum lycopersicum* cultivar ‘Moneymaker’ were sown and grown in a hydroponic medium, consisting of a modified version of the basal Murashige and Skoog (MS) salts [123] at a 0.5X concentration, and using rockwool as supporting material. In this modified medium, all sulfate salts are replaced by an equivalent concentration of potassium salts to maintain ionic balance, and the available sulfate in the medium is provided in the form of K_2_SO_4,_ in a concentration that recapitulates the original sulfate concentration of the 0.5X MS salts. The specific concentration of salts is as follows: 1.5 mM CaCl_2_, 0.625 mM KH_2_PO_4_, 3 mM KNO_3_, 0.865 mM K_2_SO_4_, 0.75 mM MgCl_2_, 50 µM H_3_BO_3_, 0.05 µM CoCl_2_, 0.05 µM CuCl_2_, 50 µM FeCl_3_, 50 µM MnCl_2_, 2.5 µM KI, 0.05 µM Na_2_MoO_4_, 15 µM mM ZnCl_2_, 50 µM Na_2_EDTA, adjusted to pH 5.7. The liquid medium was replaced twice a week to maintain constant nutrient concentrations [40]. For plants grown in sulfate deficiency conditions, K_2_SO_4_ was replaced by KCl, as previously described [40,124,125]. The additional chloride introduced (0.865 mM) is negligible compared with the basal 4.8 mM chloride present in the MS medium. Plants were maintained under controlled growth conditions in growth cabinet (Bioref-19, Pitec, Santiago, Chile) set to 22 °C with a 16/8 h light/dark photoperiod. Illumination was provided by LED lights at an intensity of 200 μmol m^−2^ s^−1^. Plants were grown for 3 or 4 weeks in sulfate sufficiency and deficiency conditions, respectively. Three independent experiments were conducted, with each biological replicate consisting of a pooled sample of 5 plants.

### 4.3. Plant Growth and Sulfate Content Analysis

Plant growth was determined by scanning leaves and roots using an Epson Perfection V600 photo scanner. Total leaf area and primary root length were determined from scanned images using the ImageJ1, version 1.54b software (https://imagej.net/ij/, accessed on 10 March 2023). Total root and leaf weights were determined using an analytical scale. Sulfate content of roots and leaves was quantified using a turbidimetric analysis [126].

### 4.4. sRNA Library Preparation and Sequencing

Tomato leaves and roots from plants grown under standard MS and sulfate-deficiency conditions were harvested and immediately flash-frozen in liquid nitrogen to preserve RNA integrity. Total RNA was extracted using the mirVana^TM^ RNA Isolation Kit (AM1560, Invitrogen, Carlsbad, CA, USA), following the manufacturer’s instructions for total RNA isolation. sRNA-Seq libraries were prepared using the TruSeq^TM^ Small RNA Library Prep Kit (Illumina, San Diego, CA, USA). Sequencing was performed on an Illumina NovaSeq^TM^ platform, generating 50 bp single-end reads.

### 4.5. sRNA-Seq Read Alignment and Differential Expression Analysis

Raw sequenced reads were quality-checked using FastQC (v.0.11.5). Adapter trimming and removal of low-quality bases (<Q20) was performed with Cutadapt [127] (v.1.15), retaining reads with a 5′ adapter and within 18–28 nt. High-quality reads were subsequently aligned to the *Solanum lycopersicum* SL4.0 reference genome using ShortStack [57,58] (v.3.8.5) with default parameters to detect and quantify the expression of sRNA clusters. ShortStack utilizes bowtie [128] to align reads with at most one mismatch, and preferring alignments with no mismatches (option —strata), and discarding all alignments for a particular read if more than 50 reportable alignments exist for it (marked as “Multi mappers ignored and marked as unmapped”). Multi-mapping reads were placed based on the local density of uniquely mapping reads in a strand-independent manner, as described in [58]. The specific arguments used were bowtie -q -v 1 -p 20 -S -a -m 50 —best —strata —sam-RG. The updated miRNA annotation was used to assign miRNA names to the identified sRNA clusters. sRNA loci identified as miRNAs (code “Y”, passing all annotation criteria included in the ShortStack 3.8.5 manual, https://github.com/MikeAxtell/ShortStack/releases/tag/v3.8.5, accessed on 25 March 2022), that were not assigned a name according to the SL4.0 miRNA annotation were classified as new miRNAs and their predicted precursor sequence was analyzed using strucVis 0.9 (https://github.com/MikeAxtell/strucVis, accessed on 10 October 2024) with default settings.

Differential expression analysis of miRNAs across experimental conditions was conducted using DESeq2 [129] (v.1.48.1), using the Wald test. The design formula used to generate the dds object was specified as design = ~ condition, allowing testing of treatment effects. All libraries were prepared, processed and sequenced together in a single sequencing run, and therefore no batch effect correction was applied. The standard results() function in DESeq2 was applied to extract differentially expressed features, without applying log fold change shrinkage. Independent filtering was carried out automatically by DESeq2 as part of the results() pipeline, using the mean of normalized counts as the filter statistic. Only clusters corresponding to miRNAs were considered for the analysis. miRNAs were considered differentially expressed if they exhibited an adjusted *p*-value < 0.05 and an absolute log_2_ fold change > 0.5.

### 4.6. RNA-Seq Read Alignment and Differential Expression Analysis

RNA-seq data from Canales et al. [40], available at the National Center for Biotechnology Information (NCBI) Sequence Read Archive (SRA) database under accession PRJNA629977 was retrieved, and reads were aligned to the *Solanum lycopersicum* SL4.0 reference genome using HISAT2 [130] (v.2.2.1). Gene-level counts were generated using FeatureCounts [131] from the Rsubread package [132] (v.2.22.1), based on the ITAG4.0 annotation. Differential gene expression analysis between experimental conditions was performed with DESeq2 [129] (v.1.48.1), as described in the previous paragraph. Genes were considered differentially expressed if they met the criteria of an adjusted *p*-value < 0.05 and an absolute log_2_ fold change > 0.5.

### 4.7. Degradome-Seq Analysis

Degradome-seq libraries for tomato were obtained from the NCBI SRA database. The cDNA sequences corresponding to tomato annotation ITAG4.0 were used to identify targets using CleaveLand [133]. Cleavage sites were defined as significant considering a cutoff *p*-value ≤ 0.05. All categories (0–4) were considered for the analysis.

### 4.8. RT-qPCR Analysis

One microgram of total RNA was treated with DNase I (Thermo Fisher Scientific, Waltham, MA, USA) to remove genomic DNA contamination, followed by cDNA synthesis with RevertAidTM RT Reverse Transcription Kit and random primers (Thermo Fisher Scientific), according to the manufacturer’s protocol. Each cDNA sample was diluted 1:4 with nuclease-free water before use. The RT-qPCR reactions were prepared using the Brilliant II SYBR Green QPCR Master Mix (Agilent), containing 10 µM of forward primer, 10 µM of reverse primer, ROX dye as a passive reference, template cDNA, and nuclease-free water, in a final volume of 20 µL per reaction. Amplification was performed on a QuantStudio^TM^ 1 Real-Time PCR system (Thermo Fisher Scientific). Raw qPCR data was analyzed using the Real-Time PCR Miner 4.0 software [134] to determine cycle threshold (Ct) values and calculate gene amplification efficiencies. The expression levels of target genes were normalized using *Actin-7* (*Solyc11g005330*) as the reference gene, as suggested in [40]. All RT-qPCR experiments were conducted with three biological replicates. Primer sequences were previously described [40].

## 5. Conclusions

Our study provides the first comprehensive temporal and organ-specific analysis of miRNA-mediated regulation under sulfate deficiency in tomato. We identified 40 sulfate deficiency-responsive miRNAs, revealing a dynamic and organ-dependent regulatory landscape. Integration of Degradome-seq data with transcriptomic analysis uncovered canonical interactions, but also expanded the regulatory space to include non-canonical targets linked to photosynthesis, redox control, protein turnover, and ion homeostasis. Interestingly, many miRNA–target pairs displayed positive co-regulation, suggesting a multilayered control and possible spatial separation of activity. Comparative analysis with Arabidopsis showed that, while a conserved core of sulfate-responsive miRNAs exists, tomato miRNAs regulate a broader functional spectrum, highlighting evolutionary diversification of sRNA networks. Altogether, these findings position miRNAs as central integrators of nutrient-, metabolism-, development- and stress-related processes during sulfate deficiency, offering new perspectives for improving nutrient use efficiency and stress resilience in crops.

## Figures and Tables

**Table 1:** Sulfate deficiency-responsive miRNAs. *Solanum lycopersicum* cv. Moneymaker seeds were germinated and grown in hydroponic medium containing modified basal 0.5 X Murashige and Skoog salts containing K_2_SO_4_ as a sulfate source (control condition) or KCl (the treatment and sulfate deficiency conditions). Three biological replicates, each consisting of a pool of five plants per condition, were used. We show the log_2_ fold change (FC) of the expression of miRNAs in the roots and leaves of tomato plants subjected to sulfate deficiency for 3 or 4 weeks, versus the control condition. Bold numbers represent significant differences obtained by DESeq2 (Wald test) in the treatment vs. control comparison, considering an adjusted *p*-value < 0.05 and an absolute log_2_ fold change > 0.5.

	3 Weeks		4 Weeks	
miRNA	Roots log_2_ FC	Leaves log_2_ FC	Roots log_2_ FC	Leaves log_2_ FC
Cardoso2018_sly-miR1128	0.88	1.08	−0.58	**1.21**
Cardoso2018_sly-miR1446-1	0.67	1.31	0.53	**3.53**
Cardoso2018_sly-miR1446-2	0.63	0.64	0.72	**3.47**
Cardoso2018_sly-miR164f	**1.10**	0.08	0.08	0.15
Cardoso2018_sly-miR167a-2	−0.62	−0.64	0.05	**−1.08**
Cardoso2018_sly-miR171b-2	−0.26	−0.18	−0.39	**−0.91**
Cardoso2018_sly-miR171f	**−1.17**	0.47	0.20	−0.15
Cardoso2018_sly-miR3627	0.30	0.73	−0.76	**1.31**
Cardoso2018_sly-miR395a-1	**3.96**	**3.93**	**4.91**	**5.65**
Cardoso2018_sly-miR395a-2	**3.71**	**3.39**	**3.83**	**6.17**
Cardoso2018_sly-miR395a-3	**3.45**	**3.29**	**5.17**	**5.44**
Cardoso2018_sly-miR395a-4	**4.02**	**3.44**	**4.26**	**5.67**
Cardoso2018_sly-miR395g	**3.96**	**3.93**	**4.91**	**5.65**
Cardoso2018_sly-miR398b	−0.82	0.02	−1.30	**−1.29**
Cardoso2018_sly-miR399a-4	−1.60	−0.07	**−4.07**	**−2.28**
Cardoso2018_sly-miR399i-1	−2.03	−0.17	−1.59	**−2.87**
Cardoso2018_sly-miR408a	−0.41	−0.71	**−1.34**	**−2.51**
Cardoso2018_sly-miR5368	0.79	0.33	1.54	**2.44**
Cardoso2018_sly-miR8032	0.39	0.55	0.16	**0.95**
Lunardon2020_sly-b2.5r1-147852_MIRNA	0.58	0.09	0.07	**1.50**
Lunardon2020_sly-b2.5r1-31088_MIRNA	−0.61	−0.79	0.10	**−1.66**
Lunardon2020_sly-b2.5r1-49254_MIRNA	**−1.35**	−0.24	−0.41	−0.77
miRBase21_sly-MIR10528	**−1.07**	0.04	−0.06	−0.61
miRBase21_sly-MIR10531	0.53	0.53	0.35	**1.36**
miRBase21_sly-MIR10539	−0.17	−0.96	−1.42	**−2.32**
miRBase21_sly-MIR167b	−0.44	−0.83	0.14	**−2.09**
miRBase21_sly-MIR169e	−1.87	**−1.95**	−1.08	**−3.40**
miRBase21_sly-MIR172b	−2.13	−0.21	0.10	**−1.27**
miRBase21_sly-MIR390a	−0.36	−0.21	0.51	**−1.21**
miRBase21_sly-MIR395a	**3.63**	**3.67**	**5.52**	**5.90**
miRBase21_sly-MIR395b	4.16	**3.10**	2.38	**6.09**
miRBase21_sly-MIR397	−0.44	−0.66	−0.55	**−2.09**
miRBase21_sly-MIR403	**−0.94**	−0.36	−0.02	−0.52
miRBase21_sly-MIR408	−0.56	−0.88	−0.70	**−2.12**
miRBase21_sly-MIR5302a	0.28	0.31	0.28	**1.60**
miRBase21_sly-MIR5302b	0.28	0.31	0.28	**1.60**
miRBase21_sly-MIR827	−1.63	0.44	−0.49	**−2.60**
miRBase21_sly-MIR9472	**1.31**	**0.74**	1.75	**3.11**
sly-b4.0r1-14493_MIRNA	1.71	**4.42**	2.64	**5.41**
sly-b4.0r1-14505_MIRNA	**4.25**	**4.62**	**6.31**	**9.19**

**Table 2:** Sulfate-responsive miRNA–target pairs. *Solanum lycopersicum* cv. Moneymaker seeds were germinated and grown in hydroponic medium containing modified basal 0.5 X Murashige and Skoog salts containing K_2_SO_4_ as sulfate source (control condition) or KCl (treatment, sulfate deficiency condition). Three biological replicates, each consisting of a pool of five plants per condition, were used. Differentially expressed miRNAs and mRNAs were determined by DESeq2 analysis comparing the treatment versus control condition. DE miRNA–target pairs were determined by analyzing publicly available Degradome-seq libraries with the CleaveLand software. A miRNA–target match was considered valid when targeting evidence was detected in at least one Degradome-seq library (*p*-value ≤ 0.05), across all degradome peak categories (0–4). U: upregulated; D: downregulated.

miRNA	miRNA Regulation	Target	Target Description	Organ	Target Regulation
miRBase21_sly-MIR10528	D_3w	*Solyc05g051230*	tRNA threonylcarbamoyladenosine dehydratase	Root	U_3w
miRBase21_sly-MIR10539	D_4w	*Solyc12g006120*	Nuclear transcription factor Y subunit B	Leaves	D_3w
miRBase21_sly-MIR10539	D_4w	*Solyc12g006120*	Nuclear transcription factor Y subunit B	Leaves	D_3w
miRBase21_sly-MIR10539	D_4w	*Solyc02g063450*	Glycine-rich domain-containing protein 2	Leaves	D_3w_4w
miRBase21_sly-MIR10539	D_4w	*Solyc10g079200*	Mitochondrial carnitine/acylcarnitine carrier-like protein	Leaves	D_3w_4w
Cardoso2018_sly-miR1446-2	U_4w	*Solyc09g091700*	2-alkenal reductase (NADP(+)-dependent)-like	Leaves	D_3w
Cardoso2018_sly-miR167a-2	D_4w	*Solyc07g008860*	WD40 repeat	Leaves	U_3w_4w
miRBase21_sly-MIR172b	D_4w	*Solyc04g049800*	AP2-like ethylene-responsive transcription factor	Leaves	D_3w
miRBase21_sly-MIR172b	D_4w	*Solyc11g073055*	Zinc finger protein ZAT11	Leaves	D_3w_4w
Cardoso2018_sly-miR395a-3	U_3w_4w	*Solyc05g053300*	dihydrolipoamide dehydrogenase precursor	Leaves	D_3w_4w
Cardoso2018_sly-miR395a-4	U_3w_4w	*Solyc09g082860*	ATP sulfurylase	Root	U_3w_4w
Cardoso2018_sly-miR395a-4	U_3w_4w	*Solyc09g082860*	ATP sulfurylase	Leaves	U_4w
Cardoso2018_sly-miR395g	U_3w_4w	*Solyc08g080050*	PGR5-like protein 1A, chloroplastic	Leaves	D_3w_4w
Cardoso2018_sly-miR395g	U_3w_4w	*Solyc10g005690*	Chloride channel protein	Leaves	D_4w
Cardoso2018_sly-miR395g	U_3w_4w	*Solyc04g054730*	Sulfate transporter-like protein	Leaves	U_3w_4w
Cardoso2018_sly-miR395g	U_3w_4w	*Solyc04g054730*	Sulfate transporter-like protein	Root	U_3w_4w
miRBase21_sly-MIR395a	U_3w_4w	*Solyc09g082860*	ATP sulfurylase	Root	U_3w_4w
miRBase21_sly-MIR395a	U_3w_4w	*Solyc09g082860*	ATP sulfurylase	Leaves	U_4w
Cardoso2018_sly-miR398b	D_4w	*Solyc03g093140*	Glycerol-3-phosphate transporter 1-like protein	Leaves	D_3w_4w
Cardoso2018_sly-miR398b	D_4w	*Solyc08g081220*	Cytochrome P450	Leaves	D_4w
Cardoso2018_sly-miR398b	D_4w	*Solyc01g067740*	Superoxide dismutase [Cu-Zn] 1	Leaves	U_3w_4w
Cardoso2018_sly-miR398b	D_4w	*Solyc02g069100*	Cathepsin B-like cysteine proteinase	Leaves	U_3w_4w
Cardoso2018_sly-miR398b	D_4w	*Solyc05g005460*	DC1 domain-containing protein	Leaves	U_3w_4w
miRBase21_sly-MIR5302a	U_4w	*Solyc12g099940*	Acyl-CoA N-acyltransferases (NAT) superfamily protein	Leaves	D_3w
miRBase21_sly-MIR5302a	U_4w	*Solyc04g077640*	Serine carboxypeptidase	Leaves	U_3w_4w
miRBase21_sly-MIR5302b	U_4w	*Solyc04g079040*	Serine carboxypeptidase	Leaves	D_3w_4w
Cardoso2018_sly-miR5368	U_4w	*Solyc06g066800*	Nucleotide-diphospho-sugar transferases superfamily protein	Leaves	D_3w_4w
Cardoso2018_sly-miR5368	U_4w	*Solyc07g045080*	2-alkenal reductase (NADP(+)-dependent)-like	Leaves	D_3w_4w
Cardoso2018_sly-miR5368	U_4w	*Solyc12g049280*	NAD(P)-binding Rossmann-fold superfamily protein	Leaves	D_3w_4w
Cardoso2018_sly-miR5368	U_4w	*Solyc09g011450*	Proteasome inhibitor-related protein	Leaves	U_3w
Cardoso2018_sly-miR5368	U_4w	*Solyc05g014130*	COP1-interacting protein 4	Leaves	U_4w
miRBase21_sly-MIR827	D_4w	*Solyc08g007800*	SPX domain-containing protein	Leaves	D_3w
sly-b4.0r1-14493_MIRNA	U_3w_4w	*Solyc09g009540*	Alpha/beta-Hydrolases superfamily protein	Leaves	D_3w
Lunardon2020_sly-b2.5r1-49254_MIRNA	D_3w	*Solyc07g065380*	zinc transporter EF026083	Root	D_3w_4w

## Data Availability

The sRNA-Seq datasets generated and analyzed during the current study are available in the NCBI Sequence Read Archive (SRA) repository, accession PRJNA1214914, available at https://www.ncbi.nlm.nih.gov/sra/PRJNA1214914, accessed on 1 August 2025. All other data generated during this study are included in this published article and its Supplementary Information files.

## Supplementary Materials

The following supporting information can be downloaded at: https://www.mdpi.com/article/10.3390/ijms26178392/s1.
