# Supplementary material for: Sulfate Deficiency-Responsive MicroRNAs in Tomato Uncover an Expanded and Functionally Integrated Regulatory Network
Source: Int J Mol Sci. 2025 Aug 29;26(17):8392. doi: 10.3390/ijms26178392 (PMC12428225; doi:10.3390/ijms26178392)
Supplement: Supplementary file 1 [file ijms-26-08392-s001.zip › FigS1_new.pdf]

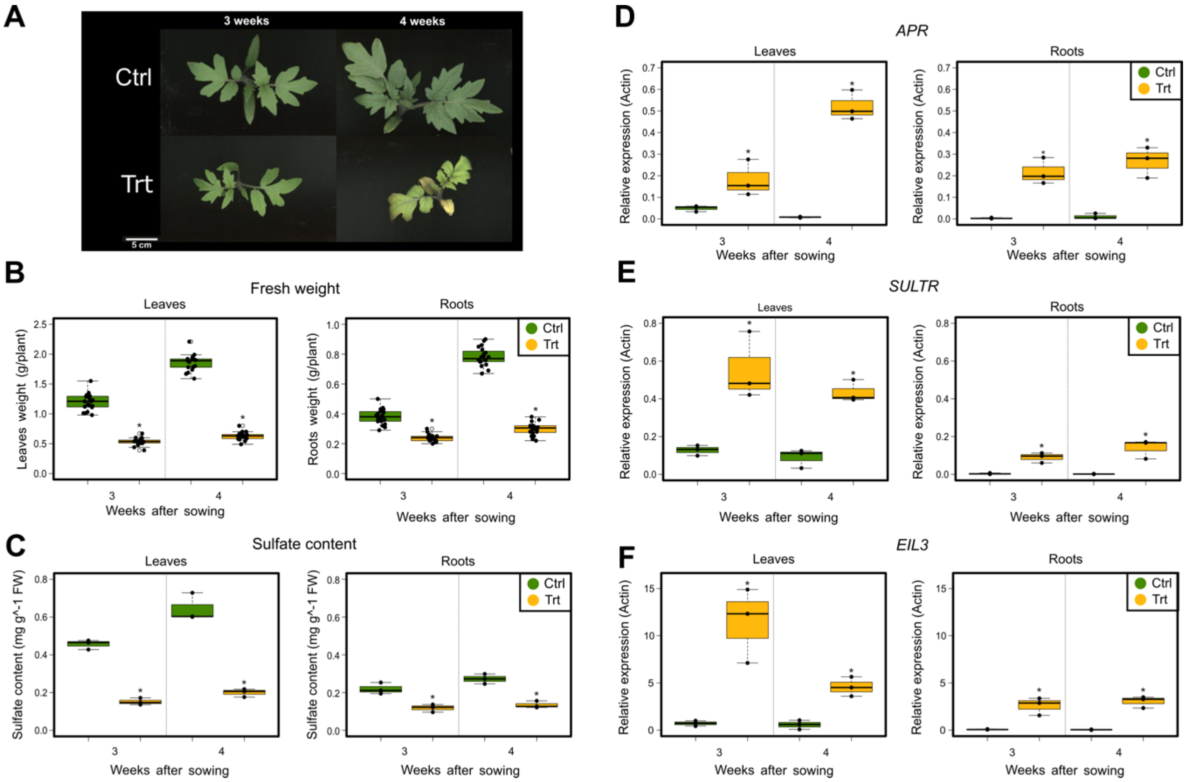

Figure S1. Phenotypic analysis of tomato plants under sulfate deficiency. *Solanum lycopersicum* cv. Moneymaker seeds were germinated and grown in hydroponic medium containing modified basal 0.5x Murashige and Skoog salts containing K_2SO_4 as sulfate source (Ctrl, control condition) or KCl (Trt, treatment, sulfate deficiency condition). (A) Growth phenotypes of tomato plants after 3 and 4 weeks of sowing under control and treatment conditions. (B) Fresh weight of tomato leaves and roots after 3 and 4 weeks of sowing in control and treatment medium. Data is representative of 15 plants (3 replicates of 5 plants each) (C) Total sulfate content of tomato leaves and roots after 3 and 4 weeks of sowing. Data is representative of 15 plants (3 replicates of a pool of 5 plants each). (D, E, F) RT-qPCR analysis of sulfur-responsive genes *APS reductase* (*APR, Soly02g080640*), (E) *Sulfate Transporter* (*SULTR, Solyc04g072760*), and (F) *Ethylene-insensitive like 3* (*EIL3, Solyc01g006650*) in tomato leaves and roots after 3 and 4 weeks of sowing under control and treatment conditions. Expression was normalized by the expression of the *Actin-7* gene (*Solyc11g005330*). Statistical comparison between control and treatment were performed using paired Student's *t-test*. Significance at p-value < 0.05 is indicated by an asterisk.
