# Supplementary material for: Sulfate Deficiency-Responsive MicroRNAs in Tomato Uncover an Expanded and Functionally Integrated Regulatory Network
Source: Int J Mol Sci. 2025 Aug 29;26(17):8392. doi: 10.3390/ijms26178392 (PMC12428225; doi:10.3390/ijms26178392)
Supplement: Supplementary file 1 [file FigS2_new.pdf › FigS2_new.pdf]

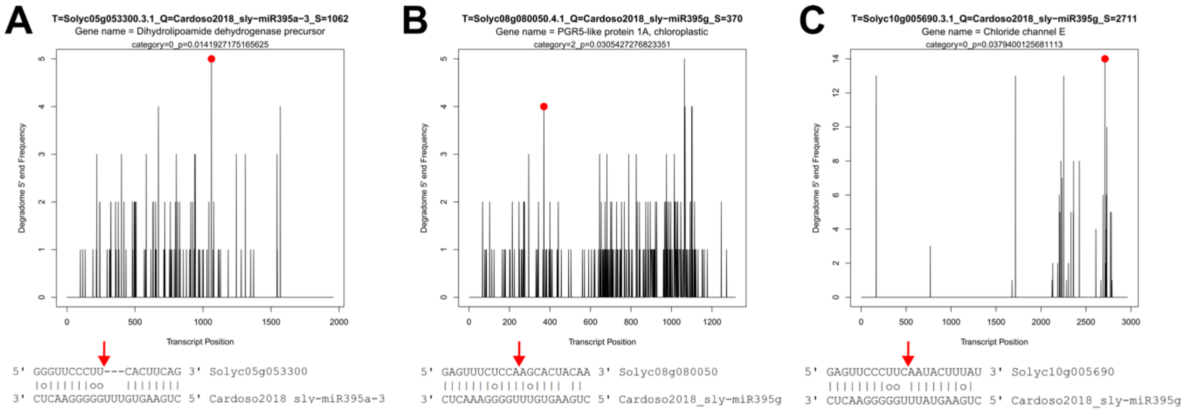

Figure S2. Degradome-seq evidence for targeting for non-canonical miR395 targets. Degradome-seq analysis was performed using mature miR395 sequences and CleaveLand with publicly available datasets. The analysis revealed three novel targets: (A) *Dihydrolipoamide dehydrogenase precursor* (*Solyc05g053300*), (B) *PGR5-like protein 1A, chloroplastic* (*Solyc08g080050*), and (C) *Chloride channel E* (*Solyc10g005690*). T-plots were generated from Degradome-seq alignments to cDNA sequences (ITAG4.0). Red dots indicate miR395-directed cleavage sites, with the corresponding sequences shown below each T-plot. Red arrows denote the cleavage position in the miRNA-mRNA interaction. Targets were filtered using p-value < 0.05 and considering all confidence categories in the CleaveLand analysis.
